# Acquisition of educational values at the Real Madrid Foundation’s social-sports schools

**DOI:** 10.3389/fpsyg.2024.1321669

**Published:** 2024-01-17

**Authors:** Gema Ortega-Vila, Enrique Ortega-Toro, Francisco Javier Giménez-Fuentes-Guerra, José Robles-Rodríguez, Francisco Alarcón-López, Manuel Tomás Abad-Robles

**Affiliations:** ^1^Real Madrid Foundation, Madrid, Spain; ^2^Faculty of Sport Science, Regional Campus of International Excellence “Campus Mare Nostrum,” University of Murcia, Murcia, Spain; ^3^Sports Performance Analysis Association, SPAA, Faculty of Sports Sciences, University of Murcia, Santiago de la Ribera, Spain; ^4^Faculty of Education, Psychology and Sport Sciences, University of Huelva, Huelva, Spain; ^5^Department of General and Specific Didactics, Faculty of Education, University of Alicante, Alicante, Spain

**Keywords:** personal and team success, self-fulfilment, personal and group superiority, health, peaceful behaviour

## Abstract

The objective of this study was to analyse the impact of the educational programme of the Real Madrid Foundation (RMF) on the acquisition of educational values at social-sports football and basketball schools among children aged between 12 and 16 years in Italy, Romania, Spain and the United Kingdom. The most significant results showed that: (a) between the 20–21 and 21–22 seasons, there were statistically significant increases in personal and team success, self-fulfilment, personal and group superiority, health and physical fitness, and peaceful behaviour; and (b) the increase in variables being studied was different according to the country analysed in such a way that: in the per-sonal and team success dimension there was a rise in the United Kingdom and Italy; in self-fulfilment there was an increase in the United Kingdom; in the personal and group superiority dimension there was a rise in Italy and Romania; in the health and physical fitness dimension there was an increase in social-sports schools in the United Kingdom and Italy; in the peaceful behaviour dimension there was a rise in social-sports schools in the United Kingdom and a drop in social-sports schools in Romania. There were very significant improvements in the majority of dimensions and countries, although the development of the various dimensions is different and specific to every one of the distinct countries, meaning that it is necessary to keep adapting the educational programme of the Real Madrid Foundation in line with the sociocultural characteristics of each of the countries in which it is implemented.

## 1 Introduction

Within teaching/learning of team sports, the new teaching/learning models based on cognitive constructivism processes and dynamic ecology, with their various approaches and distinctive features (Teaching Games for Understanding, Sport Education, Non-linear Paedagogy, Real Madrid Foundation Model, etc.) emphasise the need to create sport environments that follow certain principles of a paedagogical, physical-physiological, psychological, emotional, cognitive and social nature, in a controlled environment in order to maximise comprehensive development in children and youths (McCarthy et al., 2016; Torstveit et al., 2018; Vallence et al., 2019; Niemistö et al., 2020). A common and essential factor in all these new teaching/learning models is the need to promote values education among athletes, even designing teaching-learning models in which this aspect is the central axis, for example the “Social Responsibility Model” proposed by Hellison (2010), the “Sports Education Model” developed by Siedentop (1994), and the Real Madrid Foundation Project “For a REAL Education: Values and Sport” (Ortega-Vila et al., 2012; Ortega et al., 2021).

The Social-sports School model of the Real Madrid Foundation (RMF) “Por una Educación REAL: Valores y Deporte” (For a REAL Education: Values and Sport), is characterised by the following paedagogical approaches (Ortega et al., 2015): (a) educational values are the central axis on which the model is based; (b) inclusion and non-selection or discrimination (as regards level, gender or disability) are sought; (c) the coaches are mostly teachers; (d) the rules of the sport competition are adapted to the psycho-evolutionary characteristics of the athletes; and (e) there is an educational programme and family involvement. To implement the RMF model, regular educational activities are carried out for teachers, families and students participating in these sports schools. Likewise, the development processes of the model are decisive, through an educational evaluation that allows and favours the use of feedback and feedforward in educational matters.

The model is currently being implemented in more than 94 countries on five continents. In this respect, scientific literature stresses that it is increasingly necessary to carry out transcultural studies that make it possible to know to what extent the cultural factor of each country, territory, etc. may have an effect on generally doing sport itself (Rodríguez-Muñoz et al., 2021), on the perception that athletes have of sport values (Balish and Caron, 2015), the perception that mothers/fathers have of the important of values in sport (Bhalla and Weiss, 2010), on the relationship between values and emotions (Jin et al., 2017), the leadership style of coaches (Babbitt, 2019), on the need for coaches to adapt to the cultural environment of each country in order to achieve an appropriate education process (Borges et al., 2022), as well as on the typical effectiveness and adaptation of the different teaching models in particular (Sánchez-Gómez et al., 2014).

The objective of this study was to analyse the impact of the educational programme of the Real Madrid Foundation (RMF) on the acquisition of educational values (Personal and team success; Self-fulfilment; Personal and group superiority; Health and physical fitness; Peaceful behaviour) at social-sports football and basketball schools among children aged between 12 and 16 years in Italy, Romania, Spain and the United Kingdom.

## 2 Materials and methods

### 2.1 Participants

The sample was made up of 395 athletes from 4 countries (117 from Spain, 34 from Romania, 101 from Italy and 143 from the United Kingdom), aged between 12 and 16 years. This accounted for 60 girls and 335 boys belonging to a total of 21 RMF social-sports football and basketball schools. They had spent 1.7 ± 1.4 years on average doing sport at the RMF social-sports schools. A total of 191 athletes participated in the 2020–2021 season and 204 in the 2021–2022 season. Of the total number of athletes, 243 were novices at the RMF sports schools, meaning it was their first year, while 139 were veterans, meaning they had already participated in at least one season previously at the RMF sports schools.

### 2.2 Procedure and materials

The educational programme of the RMF was implemented which is called “Por una Educación REAL: Valores y Deporte”. This is a sport programme that places an emphasis on advancement and values education (respect, self-esteem, motivation, equality, autonomy, health and team spirit) by means of a comprehensive teaching methodology and changes to competition rules (Ortega et al., 2021). The programme was implemented for two seasons, in such a way that for 9 months each season, the athletes completed two training sessions a week, each lasting 1 h.

In order to record educational values, the Questionnaire on Values in Team Sports (CUVADE) (12–16 years) was used (Ruiz et al., 2017). The questionnaire was validated in Spanish, translated and adapted to English, Italian and Romanian. This tool comprised 29 items divided into six dimensions. Each item was answered by means of a five-point Likert scale, where 1 = not important at all and 5 = very important. The dimensions recorded in this tool are:

(a) Personal and team success (values related to the importance of winning, feeling of superiority, mastery of the sport. Example: Acting with firmness, determination and in an energetic way, in order to obtain an advantage over an opponent in a game situation);
(b) Self-fulfilment and pro-social conduct (importance given to enjoyment, self-acceptance, helping and supporting other peers. Example: Being accepted by others in the group);
(c) Sportsmanship and fair play (aspects related to respect, fairness, sportsmanship and tolerance. Example: Being sporty, polite to others and knowing how to act when winning and losing);
(d) Personal and group superiority (importance that young people give to the public image, to the feeling of superiority. Example: Feeling that we are superior to the rival team in the competition);
(e) Health and physical fitness (importance given to sport as an enabler of health and improved performance in sport. Example: Improving my fitness to improve my health);
(f) Peaceful behaviour (use of dialog as a means to resolve conflicts. Example: Acting peacefully, resolving conflicts, when they arise, through dialog).

The study was conducted in accordance with the Declaration of Helsinki, and approved by Ethics Committee (Comité Investigación Biomédica de Andalucía, PEIBA) (protocol code 0803-N-20; approved 23 July 2020).

### 2.3 Data analyses

A basic descriptive analysis of the main trends and deviations was carried out for each dimension and item, both overall and broken down into countries. The non-parametric Mann–Whitney *U*-test was used to analyse the general differences in the various dimensions between one sport season and another, making use of the rank-biserial correlation effect size.

In order to analyse the possible effect of the country in the development of educational values from one season to another, a two-way analysis of variance (2 × 4) was carried out on seasons (2020–2021 vs. 2021–2022) and country of the sports school (Italy, Romania, Spain and the United Kingdom), using the Bonferroni’s *post-hoc* test. Eta squared was employed to measure the effect size using the following values of reference (Ferguson, 2009): no effect (η^2^ < 0.04), small effect (0.04 ≤ η^2^ < 0.25), medium effect (0.25 ≤ η^2^ < 0.64), and large effect (η^2^ ≥ 0.64). All data were calculated using version 25.0 of the SPSS statistics programme, with a significance level of *p* < 0.05.

## 3 Results

Table 1 shows the means and standard deviations obtained in each of the dimensions for the 2020–2021 and 2021–2022 seasons. As regards the value ratings of the different dimensions, it was observed that the most highly valued in the 2020–2021 season were: Sportsmanship and fair play, and Health and physical fitness, while the least valued were: Personal and group superiority, Personal and team success; while in the 2021–2022 year the most highly valued was Health, physical fitness and team spirit and the least valued was Personal and group superiority.

**TABLE 1:** Mean values and standard deviation of each dimension.

	2020–2021 season		2021–2022 season		*P*-Value	Effect size
	Mean	Stand. Dev.	Mean	Stand. Dev.		
Personal and team success	3.81	0.63	4.31	0.64	0.001	0.46
Self-fulfilment and pro-social conduct	3.98	0.57	4.22	0.62	0.001	0.26
Sportsmanship and fair play	4.45	0.49	4.32	0.63	0.112	0.09
Personal and group superiority	2.73	0.92	3.46	1.08	0.001	0.39
Health and physical fitness	4.18	0.67	4.39	0.74	0.001	0.21
Peaceful conduct	3.89	0.83	4.12	1.02	0.001	0.18

When analysing the differences between the 2020–2021 and 2021–2022 years in Table 1, statistically significant increases were observed in all dimensions, except in Sportsmanship and fair play, where no statistically significant differences were found between the values of the 20–21 and 21–22 seasons.

Table 2 shows the arithmetic means obtained in each of the athlete dimensions in the 2020–2021 year, and in the 2021–2022 year, taking into account the country of the sports school of the participants.

**TABLE 2:** Mean values of each dimension, according to country of the social-sports school.

	Spain		Italy		United Kingdom		Romania	
	20–21	21–22	20–21	21–22	20–21	21–22	20–21	21–22
Personal and team success	3.78	3.75	3.95	4.55	3.67	4.67	4.33	4.01
Self-fulfilment	4.14	4.11	4.24	4.44	3.7	4.01	4.48	4.16
Sportsmanship	4.36	4.24	4.54	4.46	4.49	4.26	4.45	4.15
Personal and group superiority	2.83	2.86	3.26	4.12	2.29	3.18	3.77	3.43
Health and physical fitness	4.20	4.04	4.11	4.47	4.11	4.66	4.57	4.45
Peaceful conduct	3.96	4.00	4.17	4.38	3.63	3.94	4.5	3.75

**FIGURE 1:**
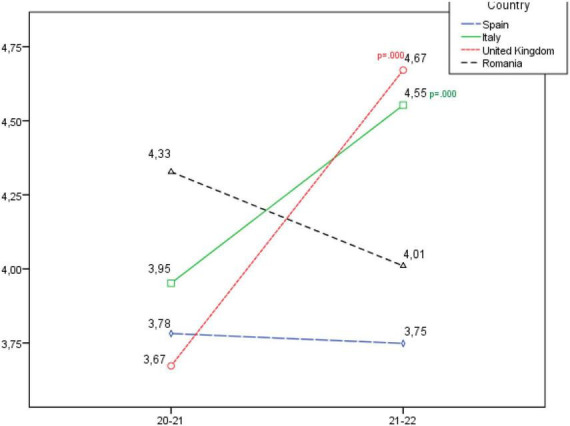
Development of the personal and team success dimension, according to country.

After carrying out a two-way analysis of variance (2 × 4) on seasons (2020–2021 vs. 2021–2022) and country of the sports school (Italy, Romania, Spain, and the United Kingdom), it was observed that the interaction effect of the Season factor with the Country was not statistically significant in the Sportsmanship dimension [*F*_(3.384)_ = 0.478, *p* = 0.689, η^2^ = 0.004]. It may therefore be stated that the interaction between both factors did not affect the changes brought about in these dimensions (see Figure 1).

On the contrary, statistically significant effects were observed in the dimensions of Personal and team success [*F*(_3.381)_ = 23.172, *p* = 0.000, η^2^ = 0.154]; Self-fulfilment [*F*_(3.382)_ = 3.644, *p* = 0.013, η^2^ = 0.028]; Personal and group superiority [*F*_(3.375)_ = 8.304, *p* = 0.000, η^2^ = 0.062]; Health and physical fitness [*F*_(3.384)_ = 6.456, *p* = 0.000, η^2^ = 0.048]; and Peaceful behaviour [*F*_(3.384)_ = 2.938, *p* = 0.033, η^2^ = 0.022].

In Figure 1 it is seen that in the Personal and team success dimension, there is a statistically significant increase in the United Kingdom and Italy, and a slight decrease in Spain and Romania which is not statistically significant.

**FIGURE 2:**
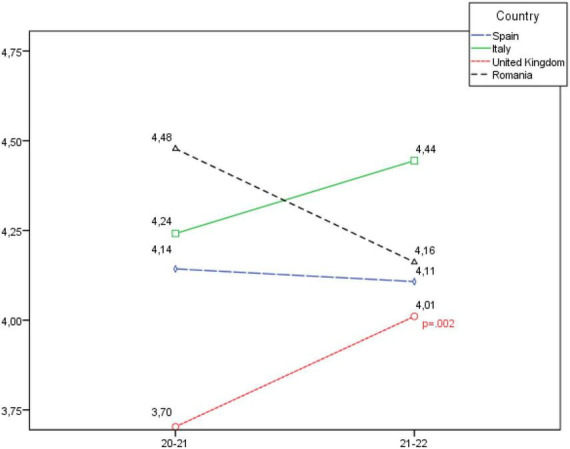
Development of the self-fulfilment dimension, according to country.

When analysing the Self-fulfilment dimension in Figure 2, a statistically significant in-crease is observed in the United Kingdom; as well as a slight increase in Italy, and a slight decrease in Romania and Italy, although in these cases there were no statistically significant differences.

**FIGURE 3:**
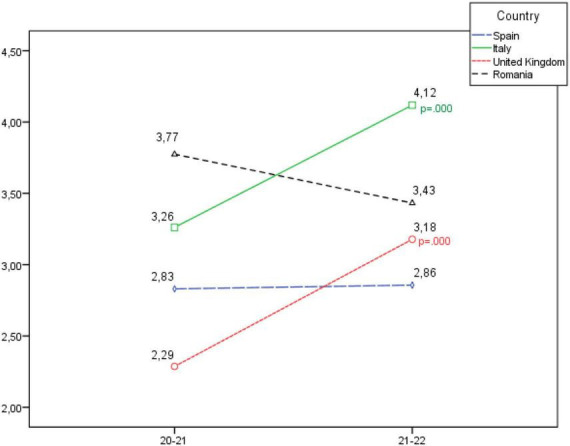
Development of the personal and group superiority dimension, according to country.

On the other hand, when analysing the Personal and group superiority dimension, in Figure 3 a statistically significant increase is seen in Italy and Romania; as well as a slight increase in Spain and a slight decrease in Romania, although in these cases no statistically significant differences were recorded.

**FIGURE 4:**
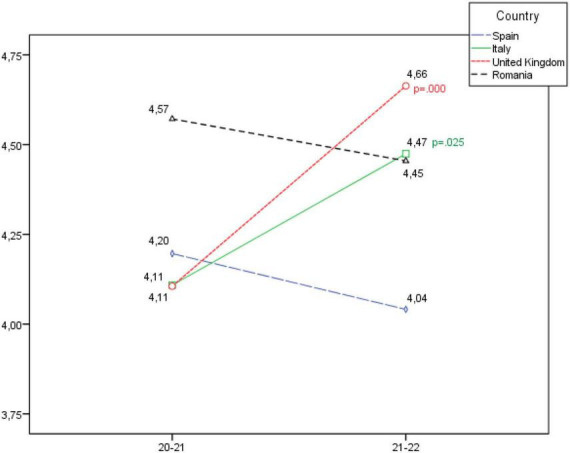
Development of the health and physical fitness dimension, according to country.

When analysing the Health and physical fitness dimension (see Figure 4), a statistically significant increase is seen in social-sports schools in the United Kingdom and Italy, and a slight decrease is observed in Romania and Spain, although in this case no statistically significant differences were recorded.

**FIGURE 5:**
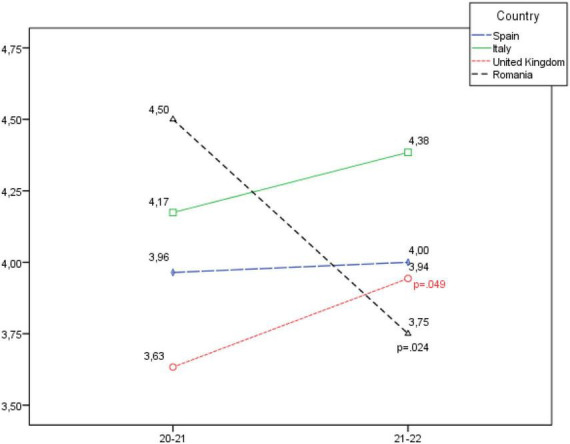
Development of the peaceful behaviour dimension, according to country.

Lastly, when analysing the Peaceful behaviour dimension (Figure 5), a statistically significant increase is observed in social-sports schools in the United Kingdom and a statistically significant decrease is seen in social-sports schools in Romania. On the other hand, there is a slight increase in Italy and Spain.

## 4 Discussion

The objective of this study was to analyse the effects of the RMF model on athlete values, considering factors of multiculturalism, according to the country where the model is implemented. However, considerable improvements are observed in the majority of dimensions and countries, while the development of the various dimensions is different and specific to the distinct countries. This aspect implies the need to keep adapting the educational programme in line with the Sociocultural characteristics.

From a general perspective, sport is an ideal environment in which to develop skills of a social and attitudinal nature (Gutierrez et al., 2017). Some current methodological approaches take these values that are normally attributed to sport for granted without observing the necessary paedagogical intention that must go with it in order for the educational experience of sport to really be valuable and positive (Shields et al., 2018; Rivera-Mancebo et al., 2020).

To date, most scientific evidence has exclusively focussed on proposals and models related to physical education in the area of curricular and formal education. In this respect, various paedagogical models in physical education have demonstrated the capacity and effectiveness as regards personal and social development (e.g., Siedentop, 1994; Hellison, 2010). In these studies, the following is observed in particular: the effectiveness of interventions for the psychosocial development of youths (Opstoel et al., 2020), improvements in behaviour related to empathy (Hastie and Sinelnikov, 2006), respect or consideration toward others (Pill, 2008), leadership (Clarke and Quill, 2003) and social relations (Molina et al., 2020). The results of this study confirm that the RMF model makes it possible to export these benefits not only to the area of formal physical education, but also to the field of extracurricular sport. Furthermore, advantages were identified in this research concerning other positive values such as health, peaceful behaviour and sportsmanship. In this respect, the current results reinforce and complete the evidence gathered in recent years about the usefulness of models based on the development of positive values.

However, just like what occurs in other studies that analyse the values of sport in different cultures (e.g., Visek et al., 2010; Jin et al., 2017), the development of these values is seen to be different according to the multicultural environment, dependent on each country. Culture can be defined as a pattern of basic assumptions, shared, invented, discovered or developed by a given group (in this case by a country), which is used to cope with its problems and which works well enough to be considered valid and, therefore, must be taught to new members of the group as the correct way to perceive, think and feel in relation to those problems (Schein, 1991). Expanding on this definition as regards doing and teaching physical activity and sport, sport coaches have beliefs and values that affect their choice of strategies, manners and ways of acting in training and teaching sessions. Any possible educational differences must be considered together with these cultural differences.

The RFM, by means of its education courses, proposes educational activities which are the basis for subsequently implementing its methodological proposals in the various social-sports schools of different countries (Ortega-Vila et al., 2012; Ortega et al., 2021). Even though the education is identical in all of them, the cultural environment has a clear effect on the way in which it is interpreted by coaches, and therefore on its implementation in the sport environment. In this sense, the results of this study show suitable developments in the different countries in almost all dimensions. Having said this, particular emphasis must be put on the improvement of the Personal and team success dimension in schools in the United Kingdom and Italy, and the decrease in values of the Health and physical fitness dimension in Spain and of the Peaceful behaviour dimension in Romania. The high data from the UK and Italy in the Personal and Team Success dimension may be attributed to the perception of both countries as traditional sports powerhouses, evident in their Olympic medal standings. Conversely, Spain has not traditionally been regarded as a nation with notable athletes or physically fit citizens, culturally placing less emphasis on this aspect and prioritising dimensions related to sports values (Serrano-Sanchez et al., 2012). Finally, low scores in the peaceful behaviour dimension in Romania could be a result of cultural and sporting influences from the former Soviet Union, where sporting success took precedence above all else (Parks, 2016).

Thus, it is suggested that the impact of the cultural environment on the development of the various dimensions of the different countries should be analysed, and also that the possibility of including cultural aspects specific to each country in the educational programmes of the RMF itself should be assessed in order to bring the model closer to the cultural needs and distinctive features of each environment.

Regarding the fact that the practical application suggests the necessity to adapt the ongoing RMF training courses conducted in various countries to the psycho-social characteristics of each nation. There is a particular emphasis on raising awareness about values with poorer indicators and generating specific strategies for coaches to implement in their respective environments. The values obtained in this study can serve as a reference so that those responsible for designing materials for the continuous training of RMF coaches can align specific proposals with the authorities in each country. Consequently, using the RMF model as a foundation, minor adaptations will be made in each country to enhance the indicators of values with suboptimal results.

The data of this study reinforce the RMF model as a cross-cutting tool applicable to any kind of cultural context, proving to be an effective alternative to the current sport models which have fed on the new social and political dynamics that are having such a negative effect on the values that have traditionally been associated with sport. Nonetheless, it shall be essential to reinforce the educational process of the model by adapting it to each of the cultural environments where it is implemented, based on the prior analysis of each cultural environment.

## 5 Conclusion

• The RMF model, a sports model that seeks to improve the values of young people through sport, is a very applicable and effective model in different cultural contexts, although minor adaptations will be necessary in each context.
• The main contributions that are appreciated in the present study of the implementation of the FRM Model are:
  ○ With respect to the improvement of the value of personal and team success the FRM model gives less importance to the results of the competition, there are no rankings, action is taken when there is too much difference to avoid large differences in the final results, etc.
  ○ With respect to the improvement of the value of self-realisation and prosocial behaviour in the FRM model, great importance is given to the athletes, both during training and competition, reaching high levels of perceived efficacy and self-realisation. For example, in competitions, many rules are modified to adapt the sport to the psycho-evolutionary characteristics of the young athletes, such as: number of participants, size of the playing field, height of the basket, etc.
  ○ With respect to the improvement of the value of health, physical fitness and fellowship, the FRM model gives great importance to the improvement of health through specific actions such as strategies to achieve adequate specific warm-ups, promote showers, improve hydration, raise awareness about the importance of changing clothes, eating fruit, or achieving an adequate diet, etc.
  ○ - Regarding the improvement of the value of Sportsmanship and fair play in the FRM model, the sportiest teams score points for white cards, coaches take advantage of conflicts to educate and promote values, it is important the union within the team and with other teams (the initial warm-up is done together, they reflect at the end of each game).
  ○ With respect to the improvement of the Personal and group superiority value and the Peaceful behaviour value, the FRM model gives great importance to the need to use dialog to resolve conflicts, so that, both in training and in competition, when a conflict arises, it is necessary to dialog to resolve it, not only individually but group actions are carried out to reflect on what happened and resolve it. For example, after the end of each game, all the players of both teams meet to comment on what happened in the game, highlighting specific actions of other players, commenting on conflictive situations so that they do not happen again, highlighting plays or actions of players, etc.
• The FRM model does something that until now had not been considered in the field of sport, which is to evaluate, plan and sequence in its plan of action in the short, medium and long term as a priority the contents related to values, in addition to other more traditional aspects such as technical, tactical, physical and psychological content.

## Data availability statement

The original contributions presented in the study are included in the article/supplementary material, further inquiries can be directed to the corresponding author.

## Ethics statement

The studies involving humans were approved by the Comité Investigación Biomédica de Andalucía (PEIBA), Código: 0803-N-20. The studies were conducted in accordance with the local legislation and institutional requirements. Written informed consent for participation in this study was provided by the participants’ legal guardians/next of kin. Written informed consent was obtained from the minor(s)’ legal guardian/next of kin for the publication of any potentially identifiable images or data included in this article.

## Author contributions

GO-V: Conceptualization, Formal Analysis, Funding acquisition, Project administration, Supervision, Validation, Writing – review and editing. EO-T: Conceptualization, Data curation, Investigation, Methodology, Writing – original draft. FG-F-G: Conceptualization, Investigation, Methodology, Supervision, Validation, Writing – review and editing. JR-R: Data curation, Investigation, Methodology, Software, Writing – review and editing. FA-L: Formal Analysis, Investigation, Methodology, Supervision, Writing – review and editing. MA-R: Conceptualization, Formal Analysis, Investigation, Methodology, Supervision, Writing – review and editing.
